# RCDB: Renal Cancer Gene Database

**DOI:** 10.1186/1756-0500-5-246

**Published:** 2012-05-18

**Authors:** Jayashree Ramana

**Affiliations:** 1 Department of Biotechnology and Bioinformatics, Jaypee University of Information Technology, 173234, Waknaghat, Solan, Himachal Pradesh, India

**Keywords:** RCC, Protein-coding, miRNA

## Abstract

**Background:**

Renal cell carcinoma or RCC is one of the common and most lethal urological cancers, with 40% of the patients succumbing to death because of metastatic progression of the disease. Treatment of metastatic RCC remains highly challenging because of its resistance to chemotherapy as well as radiotherapy, besides surgical resection. Whereas RCC comprises tumors with differing histological types, clear cell RCC remains the most common. A major problem in the clinical management of patients presenting with localized ccRCC is the inability to determine tumor aggressiveness and accurately predict the risk of metastasis following surgery. As a measure to improve the diagnosis and prognosis of RCC, researchers have identified several molecular markers through a number of techniques. However the wealth of information available is scattered in literature and not easily amenable to data-mining. To reduce this gap, this work describes a comprehensive repository called Renal Cancer Gene Database, as an integrated gateway to study renal cancer related data.

**Findings:**

Renal Cancer Gene Database is a manually curated compendium of 240 protein-coding and 269 miRNA genes contributing to the etiology and pathogenesis of various forms of renal cell carcinomas. The protein coding genes have been classified according to the kind of gene alteration observed in RCC. RCDB also includes the miRNAsdysregulated in RCC, along with the corresponding information regarding the type of RCC and/or metastatic or prognostic significance. While some of the miRNA genes showed an association with other types of cancers few were unique to RCC. Users can query the database using keywords, category and chromosomal location of the genes. The knowledgebase can be freely accessed via a user-friendly web interface at http://www.juit.ac.in/attachments/jsr/rcdb/homenew.html.

**Conclusions:**

It is hoped that this database would serve as a useful complement to the existing public resources and as a good starting point for researchers and physicians interested in RCC genetics.

## Findings

### Background

Renal cell carcinoma (RCC) represents a heterogeneous group of tumors differing in genetic background, responses to surgical and medical therapy and prognoses. It accounts for 3% of adult malignancy and results in over 100000 deaths worldwide annually [1]. It is the one of the leading causes of cancer deaths in Western countries with steadily escalating incidence over the last few decades [2]. RCC is classified based on morphological and genetic differences. This classification distinguishes metanephric adenoma oncocytoma and papillary adenoma as benign tumors from the clear cell (ccRCC), papillary/chromophilic, chromophobic (chRCC) and collecting duct RCC. This classification is important because of its prognostic implications. ccRCC is the most common and accounts for 70% of RCCs. RCC is diagnosed through imaging studies including CT and ultrasound, but kidney biopsy is an invasive technique that might result in complications and would not provide accurate diagnosis in certain situations. For early presentations, surgical extirpation through nephrectomy provides an effective treatment, but patients usually present at advanced stages, leading to poor outcomes. Even for patients without metastatic spread who undergo nephrectomy, metastatic recurrence is frequent. Apart from surgery, RCC is resistant to chemotherapy and radiotherapy. Cytokine therapy, which is reserved for patients with advanced disease, can produce partial responses in 10%–15% and durable remissions in 5% of the patients. The therapeutic approach to RCC is determined by the probability of cure, which is related directly to the stage or degree of tumor dissemination. An accurate assessment of the individual risk of disease progression and mortality after treatment is crucial to counsel patients and plan individualized surveillance protocols. Multiple studies have investigated the deregulation of genes in renal carcinogenesis at the genomic, transcriptomic as well as proteomic levels using a suite of molecular profiling techniques [3]. These include cytogenetic studies [4], gene expression analyses through tissue microarrays [5,6], serum proteomics [7], genomic resequencing [8], and microRNA profiling [9] and have yielded useful insights into RCC biology and clinical presentation, and have led to a rich understanding of the heterogeneity of this disease which greatly influences prognostic decisions. Despite the voluminous data available on RCC, the information is rather sporadic and scattered in literature.

In the last few years, a number of databases have emerged with a central focus on a particular cancer type as exemplified by Lung Cancer Database [10], Oral Cancer Database [11], Breast Cancer Gene Database [12], Cervical Cancer Database [13] etc., however there is no report of any such database for RCC. This work describes the development of the Renal Cancer Gene Database (RCDB) that catalogs the protein-coding and miRNA genes known to be involved in renal carcinogenesis as evidenced by biomedical literature. Due to its specific focus on RCC, unlike dbDEMC [14] and miR2Disease [15], it provides a far broader coverage of the miRNAsdysregulated in RCC. It incorporates information regarding the relevance of miRNAs to molecular classification of renal tumors (neoplasms) based on tumor type, metastatic status or prognosis group. This provides an additional advantage over other databases like miR2disease. Many of the protein-coding and miRNA genes in RCDB are useful prognostic and diagnostic markers and are therefore clinically relevant. These may also serve as therapeutic targets.

### Construction and content

RCDB contains information on RCC-implicated genes compiled from research articles indexed in PubMed. The PubMed database was queried with different keywords like renal cell carcinoma, renal cancer or tumor etc. and the articles retrieved were manually scrutinized to winnow the genes affecting the etiology of RCC. The final lists of 240 protein-coding and 269 miRNA genes were identified in this way and used to populate the database. The former were grouped into following six categories (Table 1) based on the kind of gene alteration observed in RCC: 1) (silencing/downregulation through) Methylation, 2) Overexpression, 3) Downregulation, 4) Mutation and 5) Translocation and 6) Unclassified. The latter (miRNAs) were categorized according to their differential expression in the different types of RCC. This classification scheme (Table 2) includes differential expression in: 1) chromocytoma vs oncocytoma 2) ccRCC vs papillary RCC 3) Poor vs Good prognosis 4) ccRCC vs normal kidney 5) chRCC vs normal kidney 6) ccRCC vs chRCC 7) Metastatic vs Non-metastatic RCC 8) Primary vs Late metastasis. There exist few overlaps of miRNAs within these categories. RCDB is implemented as a MySQL database and the web-interface built in PHP.

**Table 1:** Classification of protein-coding genes in RCDB

**Category**	**No. of genes**
Methylation	26
Overexpression	112
Downregulation	51
Mutation	24
Translocation	8
Unclassified	17

**Table 2:** Classification of miRNA genes in RCDB

**Category**	**No. of miRNAs**
1) chromocytoma vs oncocytoma	35
2) ccRCC vs papillary RCC	56
3) Poor vs Good prognosis	18
4) ccRCC vs normal kidney	190
5) chRCC vs normal kidney	57
6) ccRCC vs chRCC	64
7) Metastatic vs Non-metastatic RCC	33
8) Primary vs Late metastasis	11

### Utility and discussion

**Figure 1:**
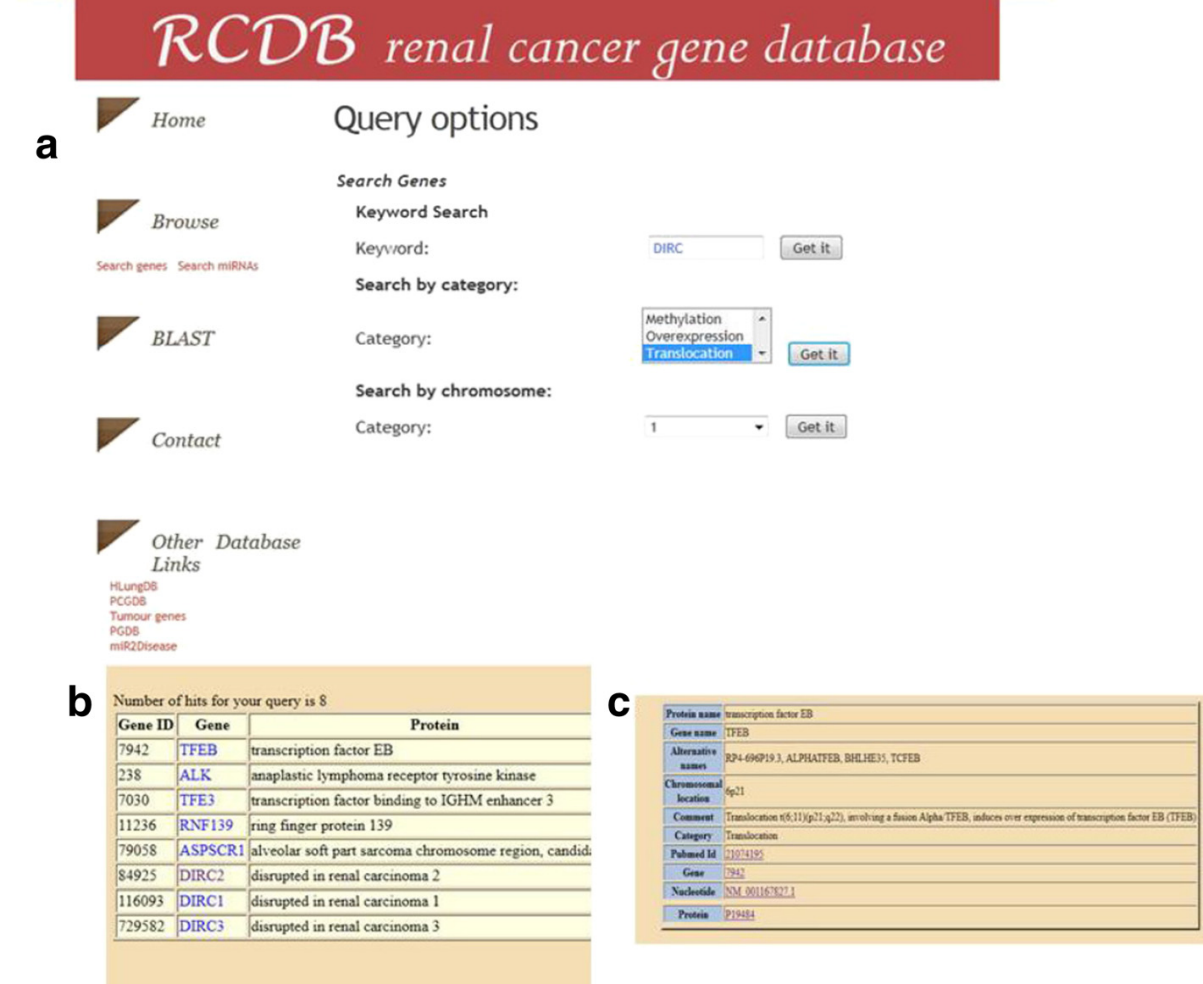
**Illustration of RCDB.** (**a**) The web interface for data query and retrieval. (**b**) The query form retrieves the list of genes as required by the query. (**c**) Each gene in the list is linked to its specific details.

The web interface query form allows users to query the protein coding genes from database using keyword, the class and the chromosome number (Figure 1a). This retrieves a list of genes (Figure 1b) where each gene entry is further linked (Figure 1c) to its specific details comprising its gene, nucleotide and protein accession numbers, its chromosomal location as well as its involvement in RCC and the PubMed records corroborating the same. The miRNAs can also be browsed in a similar way. The miRNA entries are linked to miRBase [16] wherever available. The ViroBLAST [17] tool searches a user-defined query sequence against the sequences available in the database. This offers the additional advantage of parsing the results according to E-value or score chosen by the user.

RCDB provides a comprehensive compilation of information obtained from published RCC research, complemented with the information from public databases like Swissprot, Refseq etc. It would allow the users in performing comparative studies, e.g. to deduce the genes that are shared with other cancers as well as the ones which are unique to RCC. This analysis was performed for miRNA genes in RCDB by surveying the literature for the involvement of these miRNAs in different cancers (Additional file 1). While most of the miRNAs were found to be reported in other types of malignancies, few were unique to RCC. The latter included miR455, miR219, miR509, miR627, miR648, miR510, miR379, miR136, miR376b, miR154, miR551b, miR514, miR383, miR453, miR582, miR450, miR425, miR365-1 etc.

## Conclusion

RCDB has been developed as an integrated information source to assist the research efforts of scientists and clinicians working on renal carcinoma. Besides providing a panoramic overview of RCC, it facilitates thorough exposition of each gene by providing hyperlinks to relevant PubMed records. In future, RCDB would be updated and additional data incorporated. It is anticipated that RCDB would serve as a valuable resource to the scientific community.

## Availability and requirements

Project home page: http://www.juit.ac.in/attachments/jsr/rcdb/homenew.html.

## Competing interests

The author declares that she has no competing interests.

## Author’s contributions

JR conceptualized the study, developed the database and wrote the manuscript.

## Supplementary Material

Additional file 1: A comparison of miRNAs common and unique to RCC and other cancers. Click here for file
